# Impaired Myogenic Differentiation Is a Shared Feature Across Genetic Myopathies

**DOI:** 10.3390/ijms27146338

**Published:** 2026-07-16

**Authors:** Tyler G. B. Soule, Mikhaela B. Slavin, Carly S. Pontifex, Mohamed Z. Dabaja, Arthur Melnyk, Antoine Dufour, Nicolas A. Dumont, Timothy E. Shutt, Gerald Pfeffer

**Affiliations:** 1 Department of Clinical Neurosciences, Hotchkiss Brain Institute, University of Calgary, Calgary, AB T2N 4N1, Canadacspontif@ucalgary.ca (C.S.P.); timothy.shutt@ucalgary.ca (T.E.S.); 2 Biochemistry and Molecular Biology, University of Calgary, Calgary, AB T2N 4N1, Canada; mikhaela.slavin@ucalgary.ca; 3 Snyder Institute for Chronic Diseases, University of Calgary, Calgary, AB T2N 4N1, Canada; 4 Department of Physiology and Pharmacology, University of Calgary, Calgary, AB T2N 4N1, Canada; 5 CHU Sainte-Justine Research Centre, Montreal, QC H3T 1C5, Canada; 6 Department of Physiology and Pharmacology, Faculty of Medicine, Université de Montréal, Montreal, QC H3T 1J4, Canada; 7 School of Rehabilitation, Faculty of Medicine, Université de Montréal, Montreal, QC H3T 1J4, Canada; 8 Department of Medical Genetics, Alberta Child Health Research Institute, University of Calgary, Calgary, AB T2N 4N1, Canada

**Keywords:** myopathy, adult onset, myogenesis, differentiation, autophagy, mitophagy

## Abstract

There is a shared hallmark of defective differentiation across genetic myopathies, a process that has been extensively described in Duchenne muscular dystrophy and also observed in Emery–Dreifuss muscular dystrophy. In this article, we broaden the discussion on myopathies associated with differentiation defects, examining their implications in less characterized muscle conditions that can have onset in adulthood, including facioscapulohumeral muscular dystrophy (FSHD), oculopharyngeal muscular dystrophy (OPMD), and myotonic dystrophies (DM), as well as myopathies caused by genetic variants in *FHL1*, *GNE*, *DES*, *CAPN3*, and members of the *HNRNP* family. Muscle damage can result from injury, exercise, or disease, necessitating a highly coordinated repair process to restore normal strength and function. Resident satellite cells are activated, differentiate, and fuse with the damaged tissue to facilitate this repair. This overview emphasizes the importance of muscle differentiation in the pathogenesis of myopathies with diverse etiologies and a broad range of underlying molecular mechanisms. These insights highlight differentiation as a potential convergent therapeutic target.

## 1. Introduction

Skeletal muscle regeneration is the process by which muscles adapt to physiologic stressors and respond to injury. This process is highly dependent on the function of muscle stem cells, also referred to as satellite cells, and the carefully coordinated process that results in muscle tissue repair. Increasingly, there is more and more evidence that defects in muscle regeneration are of direct importance to the pathogenesis of muscle diseases. Primary diseases of muscle are classically viewed as being a consequence of genetic defects that can result in structural alterations, abnormal aggregations, or metabolic dysfunction. In this review, we focus on the body of evidence linking skeletal muscle regeneration to the pathogenesis of late-onset myopathies.

### 1.1. Methods

This work is a narrative review with the goal of providing an expert synthesis of relevant literature and concepts. The authors of this article are content experts relevant to the subject of the article and drafted individual sections based on their specialty areas. To support each section, authors conducted targeted searches of Medline/Pubmed-listed literature using combinations of relevant keywords and Medical Subject Headings (MeSH) appropriate to their assigned topic. Reference lists from identified publications were also reviewed to identify additional sources. We prioritized original research studies from peer-reviewed Pubmed-listed literature but also included review articles where appropriate to summarize broader topics.

Because the review addresses multiple distinct aspects of the field, no predefined search strategy or uniform inclusion/exclusion criteria were applied. Evidence for citation in this review was selected based on scientific quality, contribution to the field, and the ability to highlight important concepts or controversies in the field.

### 1.2. Overview of Myogenic Differentiation

**Figure 1:**
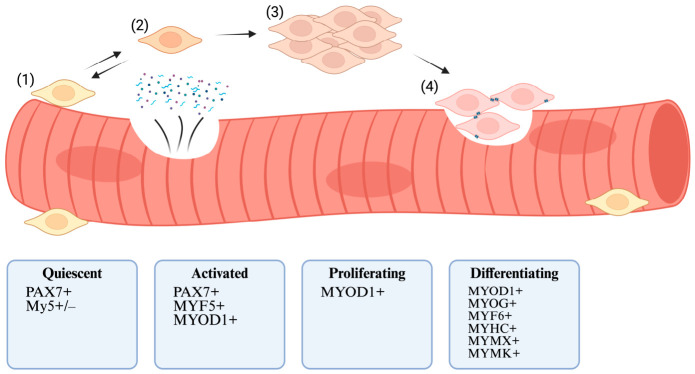
Muscle injury and repair response. Important transcription factors and proteins involved in myogenic differentiation are shown. (1) Satellite cells are quiescent. Damage due to exercise, injury, or toxins activates Pax7+ satellite cells, and they migrate towards the site of damage. (2) After expressing MYOD1, the cell can further commit or revert to its quiescent state. Cells that progress through myogenesis proliferate, forming many myoblasts. (3) This denotes the end of proliferation, where MYOD1/MYOG will push the cell towards cell cycle exit, and promote the expression of fusogen genes like Myomerger (*MYMX*) and Myomaker (*MYMK*), as well as some mature muscle genes. (4) Cells fuse with the damaged muscle, the newly added nuclei align in the center of the fiber, and their gene expression profile shifts further towards that of mature muscle.

Myogenic differentiation is the process by which muscle stem cells develop into functional and contractile muscle fibers (‘myofibers’) during regeneration. Skeletal muscle regeneration in response to exercise or injury is accomplished by the activation of muscle resident satellite cells, proliferation of myogenic progenitors, and cellular fusion to create multinucleated myotubes that are characteristic of muscle tissue. Much has been accomplished to elucidate the mechanisms behind normal myogenic differentiation [1,2]. Satellite cells express high levels of the paired box protein PAX7, reside in a quiescent state beneath the basal lamina of the muscle fiber, and are activated in response to exercise or injury [3,4]. The differentiation of satellite cells into mature muscle fibers is carried out through transient changes in the expression of myogenic transcription factors (Figure 1) [5]. Once activated, they begin to proliferate, dividing either asymmetrically to replenish the satellite cell pool or symmetrically to generate two committed progenitors. The myogenic regulatory factors, MYF5 (Myogenic factor 5), MYOD1 (Myogenic Differentiation 1), MYOG (Myogenin), and MRF4 (MYF6), are basic helix–loop–helix transcription factors that determine the progression of myogenesis. Satellite cells are either primed for differentiation or self-renewal. A small subpopulation of satellite cells expresses PAX7 but not MYF5 and symmetrically divides to expand the satellite cell pool. Satellite cells expressing PAX7 and the MYF5 transcription factor are primed for myogenic commitment [6,7]. At this stage, these cells are called myoblasts and express MYOD1, which drives proliferation and later facilitates exit from the cell cycle. MYOD1 also contacts E-boxes in closed chromatin, facilitating the opening of chromosomal architecture at numerous muscle-specific gene loci [8]. Myoblasts continue to proliferate while selectively repressing genes required for differentiation [9,10]. MYOD1 further induces the expression of MYOG, which coordinates cell cycle exit and the assembly of transcriptional machinery [11,12]. Finally, the myocytes fuse with damaged myofibers, facilitating cytoplasmic mixing and pore formation through proteins such as myomaker and myomerger [13]. This stage marks the terminal differentiation of myocytes, and the myofiber increases translation and protein synthesis, producing mature Myosin Heavy Chain (MyHC), metabolic enzymes, and upregulating mitochondrial biogenesis. Centralized nuclei migrate towards the periphery, and the myofiber physically grows until it is barely indistinguishable from undamaged myofibers [14].

### 1.3. Autophagy as a Regulator of Differentiation

**Figure 2:**
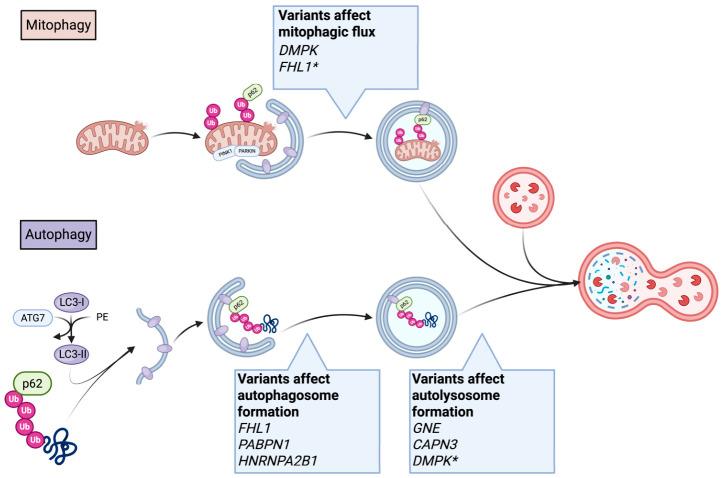
Autophagy and mitophagy in disease. The general steps of autophagy and mitophagy are illustrated. In autophagy, proteins marked by ubiquitin for degradation are recognized by p62 and guided to lipidated LC3-II in the forming autophagophore. In mitophagy, damaged mitochondria accumulate PINK1 on their surface, recruiting PARKIN to ubiquitinate mitochondrial proteins. These ubiquitin chains are recognized and enveloped. Lysosomes then fuse with the enclosed proteins or organelles, breaking them down and facilitating nutrient recycling. We mark three general stages in these processes where pathogenic variants in genes causative for myopathy result in autophagy impairments, including autophagosome formation, the fusion of the lysosome with the autophagosome, and mitophagic flux. Those genes denoted with an asterisk have a speculative relationship with the associated biological process.

Skeletal muscle is a complex tissue that is highly adaptable and undergoes high rates of cellular and protein turnover. Adequate autophagic flux is critical in both stem cells and muscle cells in quiescence, activation, and differentiation for the optimal regenerative potential of muscle in response to injury and stress, while also regulating basal tissue homeostasis [15]. We will emphasize this process, given that dysregulated autophagy is a common hallmark of many myopathies discussed later in this review. Autophagy is a highly conserved process that acts as a major catabolic system within the cell, selectively engulfing long-lived proteins, protein aggregates, and dysfunctional organelles. Specific signals that are important to autophagy are summarized in Figure 2. The general importance of autophagy in skeletal muscle is represented in many studies, with the inhibition of autophagy causing muscle atrophy, weakness, and myopathy, and impairing differentiation and regeneration [16,17,18,19]. During satellite cell activation, the expression of autophagic proteins and autophagic flux is increased [19,20,21,22]. Basal autophagy is also critical for the maintenance of the quiescent satellite cell pool prior to activation. The satellite cell-specific deletion of *ATG7*, an essential gene for autophagosome formation, enhanced the entry of stem cells into senescence and impaired the regenerative capacity of muscle [23]. Collectively, these studies indicate that autophagy is required for the maintenance of both satellite cell stemness and the activation to execute muscle differentiation. However, excessive autophagy also causes muscle wasting and impairs differentiation, establishing a requirement for the fine-tuning of autophagy for tissue homeostasis and repair [24].

### 1.4. Mitochondrial Remodeling and Differentiation

As a selective form of autophagy, the specific degradation in mitochondria through mitophagy is activated in response to physiological cues and/or mitochondrial dysfunction to support muscle regeneration and homeostasis (Figure 2). Mitochondria are labile organelles that exist as a network in skeletal muscle and are subject to a myriad of quality control (QC) mechanisms to support muscle metabolism and function [25]. In conditions of chronic muscle disuse, aging, and in muscle disease, the balance of fission (division) and fusion proteins in muscle changes to favor a molecular environment that is pro-fission [26], while exercise training promotes network elongation through fusion [27]. In this sense, mitophagy enables the adaptive remodeling of the network by removing and degrading unwanted organelles in conjunction with the activity of the fission proteins Drp1 and FIS1 [28,29]. Impairments in fission and mitophagy result in the accumulation of dysfunctional mitochondria, causing increases in ROS emissions and stimulating the release of pro-apoptotic factors to cause cell death, impairing differentiation and causing muscle atrophy [30,31]. This places mitophagy as a key mechanism driving cell survival, having significant implications for a post-mitotic tissue such as skeletal muscle.

The most extensively studied branch of mitophagy is through PINK1/Parkin, which is prominent in skeletal muscle [32]. PINK1/Parkin mitophagy is required for quiescence and the activation of satellite cells during regeneration, in addition to the basal maintenance of mitochondrial function and the homeostasis of muscle cells [33,34,35]. To this end, variants that impact mitochondrial fission and fusion can be pathogenic, impairing the adaptive plasticity and metabolic remodeling of muscle in response to injury and/or physiological challenge [36]. Despite displaying a low energetic state, quiescent satellite cells are reliant on oxidative phosphorylation, fatty acid oxidation, and are in an elongated network [37]. During differentiation, increases in fission prime satellite cells for activation and they shift towards glycolysis with a more fragmented morphology [37,38,39,40]. During terminal differentiation and maturation, they then switch back [41], as muscle is an energetically demanding tissue that primarily depends on oxidative phosphorylation [42]. Mitochondrial dysfunction may affect muscle differentiation through other processes, for example, via mitochondrial DNA release and the activation of innate immune pathways [43,44].

### 1.5. Satellite Cell Dysfunction as a Disease Mechanism in Myopathies

There is an emerging concept in the neuromuscular field suggesting that satellite cells are dysfunctional in various types of genetic myopathies, defining a new class of disorders termed satellite cell-opathies [45,46]. The importance of tightly regulated myocyte differentiation is demonstrated by the consequences to muscle function when intrinsic cellular mechanisms are disrupted. This has been most studied in Duchenne muscular dystrophy (DMD), where the absence of dystrophin protein can result in improper satellite cell polarization during division, leading to an accumulation of self-renewing satellite cells and a lack of regenerative cells [47,48]. Muscle cannot regenerate, leading to a progressive and severe myopathy. Chronic degeneration and impaired regeneration result in an early-onset severe disease which progresses to loss of ambulation, use of a ventilator to breathe, and premature death.

In this review, we explore further this new category of disease by focusing on genetic muscle diseases that can have delayed onset in adulthood, with a particular interest in genes had been investigated in the context of differentiation. Differentiation defects in the context of Emery–Dreifuss Muscular Dystrophy (EDMD) have already been reviewed [49]. Interestingly, it has become clear that a range of other myopathies exhibits impaired differentiation. Specifically, we examine FHL1opathy, oculopharyngeal muscular dystrophy (OPMD), myotonic dystrophies (DM), facioscapulohumeral dystrophy (FSHD), GNE myopathy (GNEM), Desminopathy, Calpainopathy, and HNRNPA2/B1 (Table 1). We also explore common themes among diseases, such as autophagy and mitochondrial dynamics. The presence of differentiation defects across such a wide range of genes indicates that this issue may be a more prevalent feature of myopathies than previously recognized.

## 2. FHL1opathy

### 2.1. Etiology

Reducing body myopathy was first described over 50 years ago [50]; however, it was not linked to the *FHL1* gene until 2008 [51]. Pathogenic variants in four and a half LIM domains 1 (FHL1) protein can cause a wide spectrum of diseases. Classically, variants were associated with reducing body myopathy, but the phenotypic spectrum has since expanded to include X-linked myopathy with postural muscle atrophy [52], EDMD, scapuloperoneal myopathy, and rigid spine syndrome [53]. These are collectively known as FHL1opathies, and represent a rare, clinically heterogeneous set of conditions. Onset is typically at an early age, and includes symptoms like frequent falls, contractures, distal weakness, and cardiomyopathy [54]. EDMD is inherited in a primarily X-linked fashion, with other presentations like reducing body myopathy and scapuloperoneal myopathy inherited autosomally [55]. Currently, over seven variants are known to cause EDMD, and over 50 are associated with other FHL1opathies [55]. *FHL1* variants in the LIM2 domain are pathologically distinct in that they result in menadione–NBT-positive reducing bodies [51] containing FHL1 protein [54]. These aggregates have been proposed to increase in prevalence over time [56] and are an important marker for diagnosing this condition. Also, sparing of the gluteus maximus has been reported multiple times [57,58,59,60] and could represent a useful diagnostic MRI finding. The *FHL1* gene is located on the X chromosome and has three isoforms expressed predominantly in skeletal and cardiac muscle [51]. The dominant isoform, FHL1A, is composed of an N-terminal zinc-finger domain followed by four LIM domains, each containing a double zinc-finger motif [56,61]. FHL1B and FHL1C are shorter, containing three and two LIM domains, respectively [53]. These isoforms have different interacting partners [53]. FHL1’s precise role is unknown; however, it has been shown to help coordinate sarcomere assembly, scaffold signaling proteins in the sarcomere, and regulate large transcriptional complexes in the nucleus [62].

### 2.2. FHL1 Affects Myogenic Differentiation

**Figure 3:**
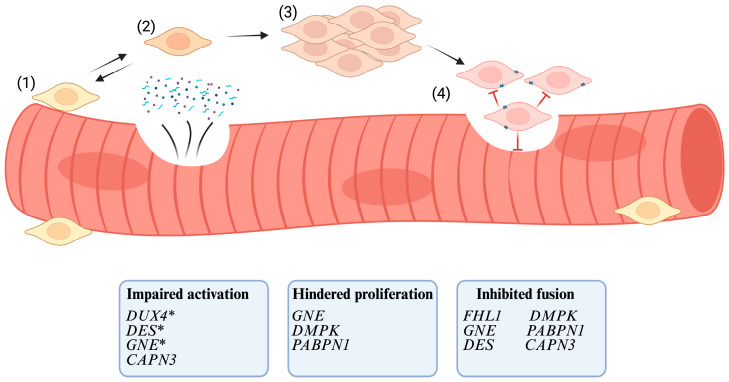
Differentiation defects for myopathies. We highlight three stages of differentiation and indicate genes where myopathic variants may interfere with a given stage. Those genes denoted with an asterisk have a speculative relationship with the associated aspect of differentiation. See Figure 1 for an explanation of steps 1 to 4. Impaired activation refers to cells which display a problem either temporally or transcriptionally in exiting quiescence (Steps 1 + 2). Myoblasts exhibiting hindered proliferation are either slower to replicate, experience cellular stress and premature senescence, or contain dysregulated myogenic regulatory factors (Steps 2 + 3). Cells with inhibited fusion have difficulty aligning and coordinating cell cycle exit, or result in myofibers with fewer nuclei (Step 4).

*FHL1* expression has a clear effect on the development and differentiation of myofibers. It is regulated by PAX7 and suppressed within hours of satellite cell activation [45]. Overexpression of FHL1 in C2C12, a spontaneously immortalized myoblast line, showed an increase in myotube size, fusion index, and expression of myogenic markers like MYOG and myosin heavy chain (MyHC) [62]. In mice, Fhl1 overexpression translated to increased strength and muscle mass [62]. One study overexpressing FHL1 in chicken myoblasts found no effect on markers of differentiation or myoblast size [63]. However, in most FHL1-related myopathy patients, FHL1 protein is reduced or absent [59,64,65,66,67]. When protein is knocked down in chicken myoblasts, fusion was inhibited and MYOG and MyHC proteins were reduced [63]. Primary myogenic progenitors from mice yielded similar findings, with fewer myofibers and lower fusion indexes in vitro [68]. This trend also occurs in C2C12 cells, with the knockdown of *FHL1* resulting in less fusion and a reduction in MYOD1, MYOG, MEF2C, and MyHC protein [69] (Figure 3). Interestingly, even with normal expression levels, disease-causing *FHL1* variants demonstrate reduced myotube area and fewer nuclei per myotube [70]. Collectively, these results suggest that the downregulation of *FHL1* may disproportionately inhibit myogenic fusion.

There has been some work aimed at elucidating the mechanistic aspect of the impaired differentiation phenotype in the context of impaired FHL1 activity. In C2C12 cells, FHL1 interacts with the Nuclear factor of activated T-cells (NFATc1), but this interaction is reduced with FHL1 variants due to the sequestration of NFATc1 in reducing bodies [62]. *FHL1* variants also affected IL-2 expression, an NFATc1 target [62]. NFATs have been implicated as important regulators in muscle, translocating to the nucleus at specific stages of differentiation to affect MyoD1 activity, influence myoblast migration, mediate myocyte fusion [71,72] and specify fiber type [73,74,75]. NFATc1, in particular, promotes hypertrophy and influences myofiber type specification [76]. Taken together, this suggests that NFATc1 may be dysregulated due to the lack of *FHL1* expression in patient muscle or its sequestration in reducing bodies. Future studies could look to see if restoring NFAT signaling could rescue fusion defects in *FHL1*-KO myoblasts.

### 2.3. Autophagy Dysregulation

*FHL1* silencing was suggested to impact autophagy, as mice showed a peak in autophagic activity in response to fasting [68]. This was extended by showing FHL1 and LC3 coimmunoprecipitating together [63]. Additionally, an accumulation of vacuolar structures was observed in cells and mouse muscle lacking *FHL1* [63,68,77], suggesting improper autophagosome formation (Figure 2). Supporting this, LC3 II/I ratios were increased in mice [68,77] and ATG5 and ATG7, genes crucial for autophagosome assembly, and were reduced in KO cell lines [63,78]. Increased expression of Beclin-3 also indicates that mitophagy could be upregulated [77]. Interestingly, NFAT has also been suggested to regulate autophagy by promoting the nuclear translocation of transcription factor EB (TFEB), and the activation of lysosomal and autophagic genes [79]. Severely affected muscle from FHL1 patient biopsies shows an accumulation of cytoplasmic and autophagic vacuoles [54]. These findings suggest that *FHL1*-KO mouse models recapitulate the autophagic defects present in human muscle and that autophagic function is impaired in both rodents and humans. Overall, there is some evidence that pathogenic variants in the FHL1 protein, or the lack of protein expression, affect the expression of NFAT and MYOG, which are crucial for autophagy and normal muscle regeneration. Future work could look to see if FHL1’s interactions with other proteins are disrupted by *FHL1* variants [80].

### 2.4. Critical Analysis

FHL1’s connection to differentiation seems to be well-supported. Based on the current literature, the loss of FHL1 impacts myogenic regulatory factor expression, and myotubes from multiple species have fusion impairments. This disruption could be due to improper NFATc1 signaling, which also influences the regulation of autophagy. However, further characterization of this pathway would be required to be confident in this assessment.

### 2.5. Possible Therapeutic Strategies

An interesting avenue for a therapeutic approach involves the protein’s closely related family members FHL2 and FHL3. Proteins in the FHL family share a similar structural arrangement, with half LIM domain followed by four LIM domains [80]. Interestingly, FHL1, 2, and 3 may have similar effects in striated muscle. In C2C12 cells, FHL2 has been implicated in the control of differentiation [81], as well as NFAT signaling [82]; it also interacts with LC3 to regulate autophagosome formation [83]. The up- or downregulation of FHL2 has a significant impact on proliferation and differentiation in bovine satellite cells [84]. It has been suggested that sparing of the extraocular muscles in dystrophies could be due to the relatively increased expression of FHL2, and that *fhl2b* expression in a zebrafish DMD model can improve survival rates [85]. Overall, FHL2 expression is significant in regulating muscle differentiation. Future work could test whether ectopic FHL2 expression could play a protective role in other DMD models and other types of dystrophies. Less is known about FHL3; however, some evidence indicates that it also determines muscle fiber type [86,87], binds Myod1 [88], and affects differentiation [89]. Notably, in mice, FHL2 is predominantly expressed in cardiac muscle, and FHL1 and 3 are expressed at different times throughout differentiation [90,91], suggesting distinct roles in this process. However, given their ability to impact differentiation through modifying expression, we propose that increasing FHL2 or FHL3 levels may represent an intriguing possibility for ameliorating differentiation defects. Although a lack of FHL1 results in reduced myogenic regulatory factor expression, it has yet to be demonstrated that restoring FHL gene family expression restores proper transcription factor activity.

## 3. Oculopharyngeal Muscular Dystrophy (OPMD)

### 3.1. Etiology

OPMD is a late-onset, progressive muscular disorder primarily affecting the muscles of the eyelids and pharynx, which may also extend to the proximal limb muscles [92,93]. Its prevalence ranges from 0.1 to one per 100,000 in Western populations. It was initially described in French Canadians [94], where the frequency is notably higher (one in 1000) due to a founder effect. Genetically, OPMD is caused by an abnormal GCN repeat expansion in the *PABPN1* gene (polyadenylate-binding nuclear protein 1), usually transmitted in an autosomal dominant manner [94]. PABPN1 plays a critical role in the post-transcriptional regulation of gene expression, particularly in mRNA polyadenylation, where it enhances poly(A) tail synthesis through direct interaction with both the nascent poly(A) tail and poly(A) polymerase [95]. The underlying mechanism of OPMD may involve a gain-of-function from the variant allele and/or a loss-of-function of *PABPN1* [96]. The *PABPN1* variant leads to the formation of toxic intranuclear aggregates that sequester nuclear proteins and RNAs, disrupting essential nuclear functions and triggering cellular defects such as impaired gene expression and apoptosis [97]. Beyond aggregation, the elongation of PABPN1 impairs its role in post-transcriptional regulation, leading to a genome-wide shift in alternative polyadenylation that predominantly impacts muscle-specific transcripts [98], probably leading to a disrupted differentiation process. The loss-of-function of *PABPN1* either because of the missing normal allele or the sequestration of the protein in nuclear aggregates was also proposed to contribute to disease pathogenesis [96]. Experiments using shRNA showed that the downregulation of *Pabpn1* increases Atrogin-1 and MuRF-1 (muscle RING-finger protein-1) expression and induces muscle atrophy [99]. Of note, it was observed that there is a more rapid decline in *PABPN1* levels in OPMD compared to normal aging, which accelerates age-associated gene expression changes, leading to premature muscle aging and impaired regenerative capacity [100].

### 3.2. PABPN1 Affects Myogenic Differentiation

*PABPN1* expression is dynamically upregulated in skeletal muscle during regeneration, suggesting an increased requirement for this protein during tissue repair [101]. During early stages of myogenic differentiation, PABPN1 interacts with the Ski-interacting protein to promote the upregulation of key transcription factors such as MyoD1 and Myog [102]. Accordingly, *PABPN1* variants reduce MyoD1 and Myog expression but also sequester Myf5 and Pax3/7 in the nuclear aggregates [103,104]. Moreover, recent studies have shown that extended PABPN1 aggregation is more pronounced in myotubes than in myoblasts [105], supporting a primary defect in cell differentiation through this mechanism. Accordingly, reduced myogenic cell fusion and premature senescence were observed in myoblasts collected from OPMD patients [106]. This differentiation defect is further supported by findings in cellular models expressing variant *PABPN1*, in which compromised fusion capacity impairs the formation of mature myotubes [107]. *Pabpn1* knockdown using siRNA or shRNA in mouse or human myoblasts significantly impairs myoblast differentiation and fusion [100,104], a process that has been linked to altered cytoskeletal spatial organization [108]. Together, these findings support a pathogenic model in which reduced and/or dysfunctional PABPN1 activity compromises muscle cell proliferation and fusion (Figure 3).

### 3.3. Autophagy Dysregulation

Emerging evidence suggests that autophagy is disrupted in OPMD. It was shown that basal autophagy is impaired in mouse myoblasts expressing the expanded form of *PABPN1* but not with *PABPN1* depletion, suggesting that the toxic gain-of-function is the underlying mechanism [109]. However, another study showed that both the downregulation of *PABPN1* or the expression of the expanded *PABPN1* variant affect autophagy-related gene expression and impair autophagic flux [110]. More specifically, the interaction between PABPN1 and HNRNPQ (heterogeneous nuclear ribonucleoprotein Q) was shown to be affected in OPMD, which alters the regulatory balance of autophagosome formation by impairing the control of ULK1 (Unc-51 Like Autophagy Activating Kinase 1) levels, a key initiator of autophagy [111]. Further study is needed to determine the contribution of autophagy dysregulation to the myogenic defects and the therapeutic potential of targeting this pathway.

### 3.4. Critical Analysis

PABPN1’s effect on satellite cell functioning and differentiation is becoming clearer. However, the impact of the pathogenic variant in OPMD is still being investigated. Protein expression increases during activation, aggregates, negatively regulates autophagy, and ultimately affects proliferation and fusion. This might occur through impaired activation as well as the sequestration of myogenic regulatory factors in the nucleus. Overall, the mechanism requires further elucidation. Particularly of interest would be determining in which context the mutation is causing a gain or a loss of function.

### 3.5. Possible Therapeutic Strategies

Given the focal onset of OPMD in specific muscles, autologous myoblast transplantation has emerged as a clinically relevant approach. Notably, myoblasts derived from muscles spared by the disease display preserved proliferative capacity in culture [106]. This observation supported the rationale for using these unaffected autologous myoblasts to reinforce compromised pharyngeal muscle function. A preclinical trial in dogs demonstrated the feasibility and safety of this strategy, with the successful integration of injected satellite cells into pharyngeal muscle tissue [106]. Building upon this, a phase I/II clinical trial confirmed the safety and tolerability of autologous myoblast transplantation in twelve OPMD patients using cells harvested from the sternocleidomastoid muscle. Improvements in swallowing function were reported, although standard imaging techniques failed to detect clear changes in pharyngeal propulsion [112]. These results demonstrate that the transplantation of myoblasts isolated from unaffected muscles could hold promise for other myopathies as well. However, this approach requires a large number of cells, and several questions remain unresolved, including whether the satellite cell pool is replenished, and the efficiency of fusion with the target tissue. Answering these questions could help to inform improved transplantation strategies. Other strategies such as gene therapy aiming to replace variant *PABPN1* with wild-type *PABPN1* showed the potential to reduce aggregate formation, reduce muscle fibrosis, improve myogenic cell survival, and restore muscle strength [113]. Altogether, these findings suggest that both cell- and gene-based approaches hold therapeutic promise for restoring the impaired differentiation capacity in OPMD muscles.

## 4. Myotonic Dystrophy

### 4.1. Etiology

Myotonic dystrophy type 1 (DM1) is a heterogeneous and multisystemic disease that is typically associated with myotonia, apathy, and progressive muscle weakness [114]. The disease can be classified according to the age of onset into congenital, infantile, juvenile, adult, or late-onset. The prevalence is around one per 8000 worldwide, making it the most frequent myopathy in adults [115]. This prevalence can reach one per 600 individuals in the Saguenay region (Canada) [116], or one per 2100 individuals in the state of New York (USA) [117]. The disease is caused by a CTG repeat expansion in the *DMPK* gene. While unaffected individuals have between five and 37 CTG repeats, this number increases to hundreds or thousands in affected individuals and is correlated with disease severity [118]. Expression of the mutated *DMPK* gene leads to the formation of nuclear RNA foci that trap RNA-binding proteins such as MBNL1 (Muscleblind-Like Splicing Regulator 1) and the overexpression of CELF1 (CUGBP Elav-Like Family Member 1), leading to alternative splicing that affects cell function, and is associated with clinical symptoms [119]. Similarly, myotonic dystrophy type 2 (DM2), which affects roughly one per 40,000 individuals, is caused by a CCTG expansion in the *CNBP* gene leading to toxic RNA gain-of-function [115]. While there are overlapping molecular and histopathological signatures between DM1 and DM2, they also differ in their pattern of muscle involvement (distal in DM1 and proximal in DM2) and the clinical manifestations and their severity [120].

### 4.2. Toxic RNA Impairs Myogenic Differentiation

The expression of *DMPK* is observed in myoblasts and is increased during differentiation [121,122]. Consequently, in DM1, there is an increase in the number of RNA foci in differentiated myoblasts compared to proliferating myoblasts [122]. Myoblasts collected from DM1 patients showed reduced proliferation and differentiation capacity [121,123], and reduced expression in MYOD1 and MYOG [123]. Upregulation of CELF1, which can bind and destabilize *MyoD1* mRNA [124], was shown to contribute to this differentiation defect [125]. The accumulation of RNA foci was also associated with increased cellular senescence and the expression of the senescence-associated secretory phenotype (SASP) [121,126,127]. Key SASP factors, such as IL-6, were associated with reduced differentiation of myoblasts in vitro, as well as impaired muscle force in DM1 patients [121,128]. Consistent with the myogenic defects observed in vitro, muscle injury in mice carrying the mutated *DMPK* gene with >200 CUG repeats resulted in reduced expression of Pax7 and MyoD, along with the formation of smaller regenerating myofibers [129]. DM2 myoblasts also exhibit signs of cellular senescence [130,131]. However, in contrast to what is observed in DM1, DM2 myoblasts do not show striking differentiation defects [132,133,134]. It has been suggested that lower levels of CELF1 in DM2 may derepress myogenic defects during differentiation [135]; however, further studies are required to clarify the underlying mechanisms.

### 4.3. Autophagy and Mitophagy Dysregulation

Muscles from DM1 patients or animal models show dysregulated expression of autophagy markers and/or impaired autophagic flux [136,137,138]. It was shown that abnormal myoblast differentiation was correlated with an increase in autophagic vacuoles and elevated levels of other autophagy markers in DM1 (e.g., LC3 ratio, ATG5) [139]. This increase in autophagy can be reduced by MBNL1 or mTOR overexpression, thereby rescuing cell proliferation [140]. However, another study indicated that the myogenic differentiation defect was independent of dysregulated autophagy [141].

Mitophagy is also dysregulated in DM1. Lower levels of mitophagy markers (e.g., BNIP3) have been observed in muscles of DM1 patients compared to controls. Signs of impaired mitophagic flux were also observed in DM2 [142]. In vitro, a reduction in mitophagic markers (e.g., PARKIN1) was observed in DM1 fibroblasts [143]. Considering the role played by mitophagy in the initiation of myogenic differentiation [144], this avenue should be further explored to explain the differentiation defect in DM1.

### 4.4. Critical Analysis

A combinatorial effect is proposed in DM1. Our current understanding suggests that RNA foci and aberrant RNA splicing interfere with proper gene expression in the cell, resulting in impaired autophagy and mitophagy, impaired MYOD1 activation, possibly through upregulation of CELF1, and an accelerated senescent phenotype. The order in which these factors come into play is unknown, as is their effects on each other. Another possible variable is the chronic inflammatory response in DM1 [145], such as SASP, which can itself impair differentiation [146]. Therefore, given the current data, it is challenging to delineate the contribution of intrinsic satellite cell deficits and altered signals from the microenvironment on myogenic differentiation impairments. The development of new inducible or conditional mouse models now makes it possible to follow the sequence of pathological events in specific cell types and determine the relative contribution of intrinsic versus extrinsic factors in regulating myogenic defects in satellite cells [147].

### 4.5. Possible Therapeutic Strategies

Several clinical trials are currently ongoing for DM1, with various strategies targeting the root cause of the disease or the clinical symptoms [148]. Among others, antisense oligonucleotides (ASOs) and antibody–oligonucleotide conjugates targeting the expanded *DMPK* RNA have been shown to rescue molecular and functional defects in DM1 muscles. In vitro, it was shown that ASOs targeting the expanded *DMPK* can restore normal splicing and reduce the number of foci in differentiated myoblasts [149]. Reduction in expanded *DMPK* transcripts by ASO was also shown to restore DM1 myoblast differentiation [150]. Alternatively, drugs may also target downstream events impairing myogenic differentiation. For instance, metformin, which targets mitochondrial complex I and stimulates mitophagy, has shown the capacity to restore cell viability [143] and muscle function in DM1 [151]. Similarly, senescence and SASP are hallmarks of DM1 that can be targeted therapeutically. Drugs such as senolytics that eliminate senescent cells (e.g., the BCL-XL inhibitor A-1155463) or senomorphics that reduce SASP expression (e.g., ASO targeting IL-6) have shown the capacity to restore myoblast differentiation [121,128]. These therapeutic advances offer promising avenues to alleviate muscle dysfunction in DM1 by targeting both upstream and downstream disease mechanisms, either alone or in combination. Notably, the connection between these therapies and differentiation is unknown, and requires deeper investigation to see if they directly impact myogenic repair.

## 5. Facioscapulohumeral Dystrophy (FSHD)

### 5.1. Etiology

FSHD is characterized by muscle weakness and atrophy starting in the face, shoulder stabilizers, and feet [152]. It progresses slowly, reaching various degrees of severity. Patients commonly report low stamina, physical fatigue, a reduced range of motion, and pain [153]. FSHD has a prevalence of between four and ten per 100,000 people [154], placing it among the most common muscular dystrophies. It appears that females have lower penetrance, as they are generally diagnosed later in life and are less severely affected [155]. In FSHD, the expression of the gene *DUX4* is recognized as the main driver of disease. *DUX4* is a retrogene, normally expressed in testis and during development; however, in most adult tissues, it is silent. When *DUX4* is not completely repressed, it is expressed in a burst-like manner in a small subset of myonuclei, making it hard to detect [156,157]. It acts as a transcription factor which regulates many other genes [158]. Its expression causes a cascade of transcriptional changes, eventually resulting in enough dysregulation that the muscle cells die [159,160,161]. However, there is evidence that this transcriptional dysregulation results in problems with differentiating myonuclei.

In FSHD1, the contraction of repeats in the D4Z4 region allows *DUX4* expression. Typically, unaffected people have between 11 and over 100 copies of the D4Z4 macrosatellite repeats, each containing a *DUX4* gene [162]. Normally, this gene is epigenetically repressed. However, in FSHD1, an abnormally low number of repeats allows for its expression [163,164,165]. FSHD2 is clinically identical to FSHD1 but is genetically distinct. In FSHD2, variants in *SMCHD1*, an epigenetic modifier, along with a permissive 4qA haplotype, results in hypomethylation of the D4Z4 chromosomal region, allowing for *DUX4* expression [166,167].

### 5.2. DUX4 Affects Myogenic Differentiation

DUX4’s widespread changes may create an unfavorable transcriptional landscape for differentiation. Notably, Jagannathan and colleagues found that most transcripts altered by DUX4 expression are consistent across in vitro models [168]. Differences arose primarily from the stage of differentiation that was analyzed. Considering that, a meta-analysis found widespread dysregulation in genes known to be affected by DUX4 [169]. Within these, many dystrophy-related genes were downregulated, such as *FHL1*, *LMNA*, *TMEM38A*, and *PLPP7*. These genes, in turn, regulate the expression of many others. For example, *TMEM38A* and *PLPP7* potentially regulate over 700 differentially expressed genes in FSHD. Among the top GO terms for these genes was differentiation. Furthermore, DUX4 represses crucial myogenic transcription factors. In C2C12 cells and mice, DUX4 suppresses *MYOD1* expression as well as other genes related to signal transduction, growth and development, and cell cycle control [161,170]. Even in cells where DUX4 target genes are not altered, PAX7 target genes are repressed [171,172]. Overall, these results suggest that DUX4 alters gene expression prior to and during myogenesis. This reinforces the unfavorable transcriptional landscape for proper differentiation in FSHD.

As DUX4 may be specifically induced during differentiation [173], it would be interesting to analyze gene expression at different stages of differentiation in the presence of DUX4. Indeed, some transcripts have already been suggested to be expressed only in the context of differentiation [168]. The fact that *MYOD1* and *PAX7* expressions are repressed in the context of elevated DUX4 activity suggests problems with the coordination of early stages of myogenesis.

### 5.3. Critical Analysis

We believe that given the breadth of transcripts altered by DUX4, including genes directly associated with PAX7 and MYOD1, it is plausible that satellite cell function is directly inhibited. However, a significant consideration is the altered expression profiles of other cell types in muscle tissue, which creates an inflammatory microenvironment [174,175], promotes FAP proliferation [176], and could inhibit differentiation [146]. Therefore, given the current data, it remains challenging to know the relative contribution of intrinsic satellite cell deficits versus altered microenvironment signals to myogenic differentiation impairments.

### 5.4. Possible Therapeutic Strategies

MATR3 (Matrin 3) demonstrates some promise in inhibiting DUX4’s action. MATR3, which binds DNA and RNA in the nucleus, can bind to DUX4’s DNA-binding domain, preventing it from promoting anomalous transcription [158]. MATR3 overexpression rescued the differentiation potential of primary FSHD myoblasts and promoted their survival. Of note, *MATR3* pathogenic variants are also associated with distal myopathy [177], FTD, and ALS [178]. However, manipulating endogenous MATR3 expression could lead to off-target effects, as the protein influences many processes such as stress granule formation [179,180], neurodegeneration [181,182,183,184], and differentiation [185,186,187]. Therefore, the authors demonstrated that an N-terminal region lacking binding domains is sufficient to restore differentiation potential in primary myoblasts. Combining this truncated protein with a muscle-specific delivery system [188] could represent a therapeutic avenue for FSHD. This approach could ameliorate intrinsic satellite cell dysfunction by removing the interference of proper transcriptional regulation while also suppressing a dysregulated niche resulting from the improper expression of extracellular matrix factors [169,189]. However, this hypothesis needs to be validated in vivo.

## 6. GNE Myopathy

### 6.1. Etiology

GNE myopathy (GNEM) was originally described in the Jewish people of Persian descent [190] and in Japanese populations as Nonaka myopathy [191]. Currently, over 150 pathogenic variants are known [192]. GNEM is inherited in an autosomal recessive manner and occurs at an approximate prevalence of 1:1,000,000; however, due to lack of awareness in clinicians and similar presentation to other myopathies, it may be underdiagnosed [193]. Heterogeneity in phenotypic outcome also compounds the difficulty in diagnosing and understanding this disease, as new conditions such as thrombocytopenia are being discovered in relation to GNEM [194,195]. *GNE* encodes the bifunctional enzyme UDP-N-acetyl−2-epimerase/N-acetylmannosamine kinase (GNE/MNK) which catalyzes the first two rate-limiting steps in the synthesis of sialic acid, an important monosaccharide involved in many biological functions [196]. GNE has a crucial role in development, highlighted by embryonic lethality when it is knocked out [197,198]. Several lines of evidence support the importance of exploring alternate roles of GNE in the cell besides sialic acid metabolism. Only 20% of GNEM disease severity can be explained by the impact that a variant has on the enzyme’s activity [199]. Variants reduce GNE enzymatic activity by 20–80%, but the phenotypic outcome for patients does not appear to correlate with a reduction in GNE activity [199]. Also, as discussed below, mixed results have been achieved by supplementing with sialic acid. GNE’s other roles could include cell adhesion, apoptosis [200], and interaction with other proteins [201,202]. The current understanding of the cellular consequences in these areas is comprehensively detailed by Pogoryelova and colleagues [203]. It is therefore worth considering that pathogenicity may not necessarily develop due to reduced sialyation, but perhaps via the disruption of secondary functional roles of GNE.

### 6.2. GNE Affects Myogenic Differentiation

New evidence links GNE deficiency with muscle differentiation defects. Schmitt and colleagues performed single-cell RNAseq on patient-derived induced pluripotent stem cells (iPSCs) and used pseudotime analysis to look at myogenic progression [204]. The patient-derived iPSCs arrested in a less differentiated state, expressing no MYOD1, MYOG, or DES. Similarly, the differentiation of mouse embryonic stem cells lacking GNE into skeletal and cardiac muscle lineages is impaired, lacking β-MyHC, PAX7, or MYOD1 [197]. *GNE* knockout in murine myoblasts or C2C12 cells resulted in slower proliferation and a complete inability to differentiate into mature myotubes, as measured by MyHC expression [205,206]. Further analysis revealed that these myoblasts were stressed, upregulating the DNA damage response both at the transcriptional and protein levels [206]. Even after supplementation with the sialic acid precursor, Neu5Ac successfully restored cellular sialylation in C2C12, and differentiation potential was not restored [205]. This suggests that differentiation defects might arise through another mechanism than a lack of sialic acid. Overall, the mechanisms surrounding impaired myogenic differentiation in GNEM are not well-understood.

### 6.3. Autophagy Dysregulation

As discussed above, autophagy is linked with myogenic progenitor activation and differentiation potential [207]. The activation of autophagy in fibroblasts with *GNE* variants through serum starvation decreased their viability. Increased autophagic flux by metformin treatment improved their viability [208]. Similar findings were demonstrated in patient-derived iPSCs, whose inability to fully differentiate correlated with transcriptional changes in autophagy [204]. Again, the pharmacological activation of autophagy with a small molecule increased the proportion of differentiated cells [204]. Notably, these results were more striking in one patient cell line than the other. However, taken together, these results suggest that modulating autophagy could rescue differentiation defects in GNEM. Further investigation into the relationship between *GNE* variants and autophagy would be key for understanding if this is a crucial impairment in muscle regeneration.

### 6.4. Critical Analysis

The importance of an alternate mechanism underpinning GNEM pathogenicity other than sialic acid deficiency is highlighted by the lack of success in clinical trials using sialic acid precursors. Although some papers suggest that myogenic regulatory factor expression is directly affected by pathogenic *GNE* variants, the mechanism is unclear, and the links remain speculative. We suggest that the limited current literature combined with the shared theme of impaired autophagy supports future investigation into the regenerative capacity of satellite cells in GNEM.

### 6.5. Possible Therapeutic Strategies

In vitro studies have demonstrated reduced enzymatic activity of both protein domains when mutated, and patients with GNEM display reduced sialylation in muscle, serum, and cultured cells [199,209]. Because of this deficiency, the primary focus of therapeutic development has been on delivering sialic acid and its precursor ManNAc (*N*-acetylmannosamine) to muscle tissue. Supplementation of sialic acid and ManNAc in mouse models carrying the GNE variants D176V and M712T abrogates the disease phenotype [210,211,212,213]. Although murine models showed promising results, these findings have not been supported in human trials. Ultragenyx halted a phase 3 trial using sialic acid after 48 weeks due to lack of statistical significance in the clinical outcome [214]. A different extended release formulation, SA-ER, has shown efficacy in preserving upper limb strength [215,216,217] in a small cohort of people with GNEM (*n* = 14). Further follow-up on the effects of SA-ER on other patient populations is required. Additionally, ManNac supplementation has shown clinical efficacy [218], and is currently undergoing a phase 3 trial [219]. If these clinical trials prove successful, it could underscore the importance of longer trials with a larger treatment group, irrespective of the disease’s rare nature. If unsuccessful, this would strongly suggest that the restoration of cellular sialic acid does not ameliorate the disease phenotype. Notably, there is no evidence to connect sialic acid availability to impaired myogenic differentiation. Therefore, other cellular mechanisms should be explored to understand which disrupted pathway leads to the impairment of differentiation.

## 7. Desminopathy

### 7.1. Etiology

Desmin myopathy causes clinical symptoms that vary depending on the mode of inheritance and specific genetic variant but typically presents in adulthood with a slowly progressive myopathy and often cardiomyopathy [220]. Desmin is the main intermediate filament expressed in cardiac, skeletal, and smooth muscle [221]. Intermediate filaments are a class of cytoskeletal proteins encoded by a family of approximately 70 genes [222]. Desmin, encoded by the *DES* gene, is responsible for anchoring and coordinating myofibrils by linking Z-disks to costameres and desomosomes, positioning mitochondria [223] and nuclei as well as participating in signaling events [224,225,226]. Anchoring myofibrils to each other and the plasma membrane allows for coordinated lengthening and shortening of the myofiber [227]. Notably, variants in the chaperone protein for desmin, the heat shock protein αB-crystallin, can cause myopathy and cardiomyopathy [228,229,230]. Given their related roles, both give rise to similar phenotypes. We focus on the role of desmin, as several reviews have linked αB-crystallin and differentiation [230,231,232].

The main hypotheses for desmin’s role in pathogenesis include signal transduction, stabilization of RNA, and differentiation [233]. The idea of inhibited differentiation is not new [233]; however, there has been progress in the mechanistic understanding of desmin’s role in this process. In vitro studies suggest the indispensable nature of desmin in development. Specifically, embryonic stem cells lacking desmin were unable to form smooth or skeletal muscle [234]. In vivo studies reveal that desmin does not appear to be required for the formation of skeletal or cardiac muscle during development [233,235]. However, within two weeks post-natal, its absence results in a myriad of defects, including disorganized myofilaments, centralized nuclei, and eventually fibrosis [233,235]. This translates to functional strength deficits after 3–4 months [236]. These studies observed muscle group-specific changes, with the relative preservation of function in other anatomical regions, which has been attributed to differential muscle usage patterns [233]. This explanation is plausible, as fibers lacking desmin are more susceptible to damage due to contraction [235]. Overall, in murine models, desmin is not required for developmental myocyte fusion and contractile apparatus formation; however, muscle defects become apparent shortly after birth.

### 7.2. Desmin Affects Myogenic Differentiation

Desmin is expressed in satellite cells [237] and is upregulated very early in the differentiation process by the MEF2C transcription factor [238]. In vitro culturing of primary cell lines from patients with the DES L345P variant showed that disruptions in desmin networks only emerge after extended periods of culturing [239]. Early passage primary cells with the CRYAB R120G variant, causing desmin aggregation, showed that aggregation only occurred after a differentiation stimulus was applied [240]. These findings could allude to desmin’s importance only after the initiation of differentiation. In murine skeletal muscle, satellite cell number and Pax7 expression are unaffected in desmin null mice, but markers of differentiation like MyoD1, Myog, and embryonic MyHC are reduced [241]. Additionally, ectopic expression of desmin in murine myoblasts lacking emerin or A-type lamins restores differentiation potential without upregulating MyoD1 [242]. This finding suggests that desmin could regulate the expression of some myogenic transcription factors. Future studies could validate if desmin can rescue differentiation defects in other disease conditions or if primary human myoblasts downregulate myogenic transcription factors in response to the lack of desmin. Another important aspect of proper differentiation is the niche surrounding satellite cells, including interaction with the extracellular matrix [2]. A recent study found that desmin R405W knock-in mouse satellite cells form fewer focal adhesions within muscle and migrate faster [243]. Coupled with the previous findings of defective repair, this observation suggests that desmin variants might impair satellite cells’ ability to properly migrate to the site of muscle damage.

### 7.3. Nuclear Stability Promotes Differentiation

Desmin has a prominent role in nuclear stability and may act as a mechanosensor by communicating force to the nucleus [244,245]. Its role in anchoring nuclei is well characterized. Ablating desmin results in the loss of nuclear shape, integrity, and cellular distribution [246]. It has been shown to interact with other nuclear proteins like lamin B [245,247,248]. Taken together, desmin has an established link to nuclear function. Differentiation defects are mirrored by other proteins with nuclear contacts. The disruption of nuclear-interacting proteins such as LAP1B (Lamina-associated polypeptide 1B), Lamin A/C, and RUNX1 is pathogenic to muscle. Loss of *LAP1B* causes similar defects in myoblasts, including impaired differentiation, limited fusion, and extracellular matrix disorganization [249]. *LMNA* encodes Lamin A/C, a part of the linker of nucleoskeleton and cytoskeleton complex (LINC) [250]. Lamin A/C is upregulated in cycling myoblasts [251], and variants in *LMNA* can manifest as EDMD [252]. RUNX1 serves to modify chromatin structure and acts as a nuclear scaffolding protein [253]. If overexpressed in C2C12 cells, it inhibits myogenic differentiation [254]. Taken together, altered function in nuclear players is linked to impaired differentiation. Given desmin’s involvement in nuclear signaling, this could be an avenue through which differentiation problems arise. Overall, desmin is required for normal function after development and could regulate key myogenic transcription factors through its interaction with the nucleus. Future work is needed to define the extent to which desmin variants alter nuclear dynamics and whether that results in a modified transcriptional program. Restoring myogenic transcription factors like Myod1 in the absence of desmin would be an interesting test for whether desmin’s role in transcriptional regulation is responsible for defects in differentiation. Additionally, it would be important to validate whether these findings translate to human myoblasts.

### 7.4. Mitochondrial Dynamics

The importance of mitochondrial network morphology for organelle function has been discussed in previous sections. Desmin additionally supports the contractile function of muscle by maintaining the structural integrity of the mitochondrial reticulum, with evidence showing that impaired function of desmin negatively impacts the ability of mitochondria to produce ATP [255,256]. Variants in desmin can promote the formation of intracellular protein aggregates, impairing sarcomeric organization and having negative effects on muscle function [257]. However, both aggregate-prone and nonaggregate-prone variants caused impaired mitochondrial morphology and function in muscle [258]. In particular, desmin variants caused fragmented mitochondrial networks and abnormal organelle distribution, including the accumulation of subsarcolemmal mitochondria [224,233,258], yielding reduced membrane potential and respiration [259]. These impairments in mitochondrial function additionally impeded the ability of the organelle to maintain the genome, culminating in reductions in mtDNA copy number and an increased number of deletions [223,258,259,260]. As discussed in a previous section, mitochondrial instability in desminopathy could lead to mtDNA release, propagating inflammation and contributing to the pathological phenotype. Despite the role of desmin in mitochondrial organization being well-established [261], uncertainty remains with regard to the mechanisms of dysregulated mitochondrial morphology under disease conditions. However, some evidence points to aberrant levels of activated Drp1 to favor a pro-fission cellular environment and a fragmented network. Many mechanisms regulate the activity of Drp1, including phosphorylation, S-Nitrosylation, SUMOylation, and ubiquitination [262]. Drp1 can be recruited to mitochondria by changing the phosphorylation status of Ser-616 or Ser-637 [262,263]. Overexpression of desmin with a seven amino acid deletion in the mouse model led to the elevated expression of fission proteins including the activating phosphorylation of Drp1 at Ser-616 [259], generating a disruption in the fission–fusion balance. Moreover, the inhibition of fission in this model attenuated cardiomyocyte death and improved contractility, connecting the regulation of mitochondrial dynamics and cardiac function [259]. Along with a fragmented mitochondrial network, elevated Drp1 promotes intermediate filament disassembly through the phosphorylation of desmin at Ser-31. This post-translational modification increases the vulnerability of desmin filaments to form aggregates [264,265] and acts as a pathological hallmark in desminopathy. Changes in mitochondrial morphology by Drp1 are connected with reductions in mitochondrial transport [266], suggesting changes in the interactions between kinesin proteins and mitochondria. Kinesin proteins are ATP-dependent motor enzymes responsible for the anterograde (outward) transport of mitochondria on microtubule tracks, typically to cellular areas with high ATP demand. Kinesin proteins are known to play an important role in differentiation, as enhancing their activity was able to restore differentiation and the regeneration of muscle in aged animals [267]. Importantly, when desmin is knocked down in mice, kinesin and mitochondria lose contact, concurrent with a change in the distribution and function of mitochondria [268], potentially contributing to changes in muscle function in desminopathy [223]. Giovarelli and colleagues observed increased Ser-616 phosphorylation and Drp1 recruitment to mitochondria in the presence of desmin aggregation [264], driving mitochondrial fission. However, many kinases and regulators have been identified to act on Drp1 in specific contexts [262,269]. Drp1 and other fusion and fission proteins have also been reported to be misregulated in BAG3 (Bcl2-associated athanogene 3) myopathy [270] and *EMD* (Emerin gene) silencing [271], both of which can also affect cardiac tissue. Since Drp1 is crucial for myogenesis, abnormal Drp1 expression combined with dramatically altered mitochondrial dynamics could impair the proper activation of satellite cells or fusion of myocytes in desminopathy, blunting the regenerative capacity of muscle. An interesting line of future research would be to investigate Drp1 expression and localization during satellite cell activation and over the course of differentiation in desminopathy. Also, it would be of interest to elucidate how Drp1 is regulated in the context of desminopathy to drive changes in mitochondria, differentiation, regeneration, and overall muscle function. However, given that desminopathy is a multifactorial disease, targeting mitochondrial dynamics in isolation may not be sufficient to improve pathological defects.

### 7.5. Critical Analysis

Desmin variants have been studied extensively and there are clear lines of evidence linking desmin with differentiation, including expression in satellite cells, upregulation with differentiation, and the compensatory ability to restore differentiation in other disease models. Possible mechanisms underpinning the differentiation defect include loss of signal transduction to the nucleus and a fragmented or damaged mitochondrial network. It is plausible that all these mechanisms play a key role in impaired differentiation. However, these connections remain speculative and require validation.

## 8. Limb Girdle Muscular Dystrophy Type 2A/R1 (LGMD2A/R1)

### 8.1. Etiology

Calpain-3 (CAPN3) is a muscle-specific, non-lysosomal cysteine protease that plays essential roles in sarcomeric remodeling, myofiber homeostasis, and muscle regeneration [272,273,274,275]. The *CAPN3* gene is predominantly expressed in skeletal muscle tissue. Unlike the ubiquitously expressed calpain-1 and -2, CAPN3 has a unique autolytic activation mechanism and contains NS, IS1, and IS2 domains, which confer muscle-specific functions [276,277]. Either a single variant or several variants in the *CAPN3* gene can cause Limb–Girdle Muscular Dystrophy (LGMD) Type 2A/R1 (LGMD2A/R1), the most common form of autosomal recessive LGMD. Over 500 distinct variants in CAPN3 have been reported, encompassing missense, nonsense, splicing variants, and small deletions [278,279]. The mean age of onset is in people of twenty years of age, and over 80% of patients require a wheelchair before they are fifty years old [280]. Pathogenic variants often result in unstable or non-functional CAPN3 proteins, impairing its proteolytic activity and disrupting key homeostatic functions in muscle fibers. Clinically, LGMD2A/R1 is characterized by progressive weakness in the pelvic and shoulder girdle muscles, typically beginning in adolescence or early adulthood. Some patients show pseudo-metabolic features, such as cramps and elevated CK levels, before weakness becomes clinically apparent [281]. Histologically, muscle biopsies often exhibit variations in fiber size, central nuclei, and an abnormal accumulation of sarcomeric and cytoskeletal proteins, indicating impaired proteostasis [277].

### 8.2. Calpain-3 Affects Myogenic Differentiation

The role of CAPN3 in skeletal muscle differentiation is being studied more as evidence suggests its involvement in myoblast fusion, myonuclear positioning, and the regulation of muscle stem cell fate [279]. In in vitro models, the silencing or genetic deletion of CAPN3 causes delayed myotube formation, abnormal myoblast alignment, and a lower fusion index, showing that CAPN3 is directly necessary for terminal myogenic differentiation [278,281] (Figure 3). Mechanistically, CAPN3 interacts with structural proteins such as titin and filamin C, and it influences the dynamics of the cytoskeleton during myoblast elongation and alignment [276]. CAPN3 has been shown to cleave key regulatory proteins, including calcineurin A, which affects the NFAT signaling pathway critical for differentiation [282]. Additionally, CAPN3 deficiency leads to the buildup of p53 and cell cycle inhibitors, likely due to a proteolytic dysregulation, which contributes to defective cell cycle exit and differentiation [276]. Importantly, recent transcriptomic data from CAPN3-deficient (*Capn3*^−/−^) mouse muscle show the impaired activation of MyoD1 and Myog, and increased Pax7 expression, indicating that satellite cells stay in an undifferentiated state [283]. This was also detected in in vivo studies as the regenerative capacity was significantly reduced after injury in *Capn3*^−/−^ mice [284]. Interestingly, the role of CAPN3 in nuclear positioning during myofiber maturation appears essential. *Capn3*^−/−^ myotubes display abnormal nuclear clustering and disrupted orientation along the fiber axis, affecting contractile function. These nuclear abnormalities, reported in both in vitro and in vivo models of CAPN3 deficiency, are likely caused by defective cytoskeletal organization and impaired tension sensing during myofibrillogenesis. This highlights the important role of CAPN3 in shaping the spatial organization of multinucleated muscle fibers, a key factor for proper muscle mechanics and regeneration.

### 8.3. Autophagy Dysregulation

CAPN3 also plays a key role in proteostasis and mitochondrial function, and its deficiency leads to a multifaceted disruption of cellular quality control. In *Capn3*^−/−^ muscle fibers, studies have identified aberrant accumulation of ubiquitinated proteins, suggesting the impaired turnover of damaged components via the autophagy–lysosome system [285]. CAPN3 is additionally thought to act upstream of autophagic flux regulation, possibly by cleaving key substrates that are not yet fully characterized and involved in vesicle maturation and fusion. The *Capn3*^−/−^ muscle exhibits elevated LC3-II levels and the accumulation of p62/SQSTM1, indicating a block in autophagosome clearance, rather than increased autophagy initiation [286]. This may compromise the cell’s ability to clear damaged organelles and protein aggregates, contributing to chronic stress and atrophy.

### 8.4. Mitochondrial Dynamics

Mitochondrial dysfunction is another important key outcome of LGMD2A/R1. *Capn3*^−/−^ mice were demonstrated to have impaired mitochondrial networks, decreased expression of oxidative phosphorylation (OXPHOS) complexes, and dysfunctional ATP production [287]. These effects may be mediated through the secondary activation of stress signaling pathways such as AMP-activated protein kinase (AMPK) and forkhead box O3 (FOXO3), which are known to regulate both autophagy and mitophagy [288]. Calpain-3 has also been implicated in the regulation of mitophagy through the BNIP3 pathway and the PINK1 and E3 ubiquitin–protein ligase Parkin signaling axis [289]. The accumulation of dysfunctional mitochondria in CAPN3-deficient fibers likely contributes to oxidative stress and exacerbates the degenerative phenotype. Additionally, electron microscopy of patient biopsies and mouse models shows enlarged and structurally damaged mitochondria, often found near Z-disks or areas of sarcomeric disruption [290]. These structural changes are linked to decreased endurance and exercise capacity, further connecting CAPN3 to muscle energy metabolism.

### 8.5. Critical Analysis

There is a considerable body of evidence connecting loss of CAPN3 with the direct inhibition of myogenic transcription factors, possibly through Calcineurin A’s impact on NFAT signaling. Also reported are increases in cell cycle inhibitors and functional deficits in the initiation of differentiation and fusion. It seems reasonable that the disruption of nuclear positioning, mitochondrial turnover, and autophagic activity could all contribute to these deficits; however, there is insufficient mechanistic evidence to support a causal relationship.

### 8.6. Possible Therapeutic Strategies

Therapeutic efforts have been mostly focused on gene therapy, modulation of proteostasis, metabolic reprogramming, and stem cell-based interventions. One of the most promising therapeutic strategies is the AAV-mediated gene replacement therapy, although CAPN3’s large coding sequence (~2.4 kb) and autoproteolytic activity represent challenges. Recent approaches using dual-AAV vectors or truncated, hyperstable CAPN3 constructs have shown encouraging results in *Capn3*^−/−^ mice [291]. Another option is the editing of CAPN3 variants in satellite cells or myoblasts. While CRISPR approaches are in early stages, in vivo base editing may offer the ability to correct common pathogenic variants with high specificity and minimal off-target effects [292]. However, challenges related to immune compatibility and in vivo engraftment currently remain a hurdle. Modulating the downstream consequences of CAPN3 deficiency represents a promising therapeutic option. For example, the pharmacological activation of proteolytic systems using inhibitors such as rapamycin or spermidine [293,294] with metabolic reprogramming using AMPK activators or PGC1α [294,295] may help restore proteostasis in CAPN3-deficient muscle [295].

## 9. HNRNPA2/B1 and HNRNPA1

### 9.1. Etiology

Heterogeneous nuclear ribonucleoproteins A2/B1 (*HNRNPA2B1*) and heterogeneous nuclear ribonucleoproteins A1 (*HNRNPA1*) cause multisystem proteinopathy (MSP) [296], formerly known as inclusion body myopathy with Paget Disease of Bone and/or Frontotemporal Dementia [296]. We chose to discuss these two proteins together because of their high functional and structural homology, which likely arises from an early gene duplication, as well as a number of parallels that exist between them regarding pathogenic variants and their associated clinical phenotypes [297].

HnRNPA2B1 is a regulator of alternative splicing and nonsense-mediated mRNA decay [298]. It was further identified as a mediator of N6-methyladenosine-dependent (m6a) nuclear RNA processing events [299]. m6A is a co-transcriptional modification of RNA that is prevalent in eukaryotes [300]. One third of all RNA carries 3–5 m6A modifications, which are recognized and bound by m6A RNA-binding proteins, or ‘readers’ such as hnRNPA2B1 and hnRNPA1 [300]. m6A readers participate in miRNA processing, alternative splicing, and RNA structure switching [300]. Thus, the presumed disease mechanism could be related to changes in RNA regulation and m6A activity. Some emerging evidence suggests that these proteins are important for myogenic progression. Here, we attempt to reconcile how impairments in myogenesis might be connected to the better-known functions of these proteins, such as cytosolic stress granule formation and transcriptional regulation within the nucleus.

### 9.2. HNRNPA2/B1 Affects Myogenic Differentiation

HnRNPA2B1 regulates myogenesis and myogenic fate, where stemness, cell polarity, motility, cell–cell adhesion, and differentiation must come into play [301]. hnRNPA2B1 and hnRNPA1 expressions are elevated in differentiating and proliferating mouse muscle satellite cells, where hnRNPA2B1 behaves as a myogenic splicing regulator [301]. hnRNPA2B1 protein increases during the differentiation of myocytes but tapers off during myotube formation [301]. While hnRNPA2B1 knockout myoblasts can differentiate, they fail to form large multinucleated myotubes [301]. Similar findings were identified in bovine and murine muscle satellite cells where cell fusion was impaired with decreases in hnRNPA2B1 expression [301,302]. It was determined that this impairment was mediated by bta-miR-206 and a novel lncRNA, lncA2B1, which binds to hnRNPA2B1 [302]. Additionally, frameshifted hnRNPA2 caused the apoptotic cell death of differentiating C2C12 mouse myoblasts [303]. Together, this suggests that hnRNPA2B1 activity in the nucleus is necessary for fusion. Therefore, the downregulation of hnRNPA2B1 appears to lead to failed multinucleation.

### 9.3. Cytotoxic Stress Granules or m6A Nuclear RNA Processing Events as a Putative Disease Mechanism

**Figure 4:**
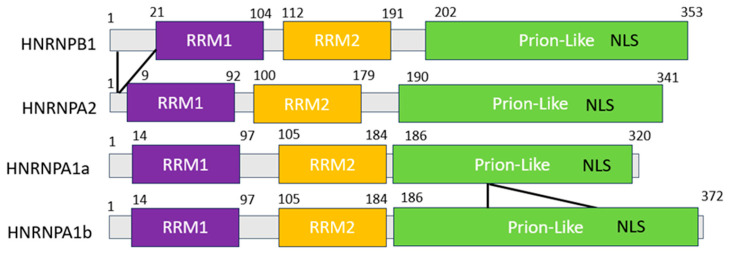
Domain architecture of classical MSP genes and hnRNPA2B1 and hnRNPA1. hnRNPA2B1 and hnRNPA1 contain two RRM domains and a C-terminal prion-like disordered domain. Within the prion-like domain is a nuclear localization signal (NLS) [305]. hnRNPA2B1 has long (hnRNPB1) and short (hnRNPA2) isoforms that differ by 12 amino acids. HnRNPA1 also has two isoforms that differ by 51 amino acids in the prion-like domain for the long (hnRNPA1b) and short (hnRNPA1a) isoforms.

Interestingly, there are structural elements that could also plausibly contribute to the disease mechanism. As RNA-interacting proteins, hnRNPA2B1 and hnRNPA1 contain RRMs (RNA Recognition Motifs), which bind to target RNA [297,304]. They also contain c-terminal prion-like disordered domains, which facilitate stress granule formation [305]. Genetic causes of neuromuscular disease like ALS and inclusion body myopathies have been frequently associated with RNA-interacting proteins with prion-like domains [305,306,307,308,309], and pathogenic variants producing insoluble cytosolic stress granules [310,311,312]. This holds for hnRNPA2B1 and hnRNPA1, with pathogenic variants tending to occur in either the prion-like disordered domains or the nuclear localization signals of the protein [313,314]. Normally, both proteins localize to stress granules as a part of the cellular stress response [315]. In *S. cerevisiae*, *Drosophila*, and humans, variants in hnRNPA2B1’s prion-like domain tend to form pathologic cytosolic aggregates [313,316,317]. In humans, the dominant wild-type isoform of hnRNPA2B1, hnRNPA2 (Figure 4), was shown to localize to the nucleus, while the pathogenic D290V variant is recruited to stress granules and accumulates in cytoplasmic inclusions [313]. The hnRNPA1 variant p.P288S/P340S also accumulates in the cytoplasm while controls only exhibit nuclear localization, which is similar to outcomes of ALS-associated variants of stress granule proteins FUS and TDP-43, where stress granule aggregates are observed to persist in the cytoplasm of neurons [306,318]. Therefore, relevant questions to answer would be as follows: Do stress granules promote impaired nuclear m6A alternative splicing and RNA processing events by segregating hnRNPA2B1 and hnRNPA1 in the cytosol, where they are unable to process RNA? Could these RNA processing events in the nucleus be necessary for myogenic progression and be the cause of impaired myogenesis?

Notably, disease progression is much more aggressive when the effect of the variant does not directly lead to increased propensity for fibrile formation, but rather impacts the nuclear localization sequence, thereby impairing nuclear import [303]. hnRNPA2B1 frameshift-variants produce a particularly aggressive early-onset OPMD. Unlike the D290V variant in the prion-like domain, the frameshift-variants do not inherently increase fibril formation, instead impacting the nuclear localization signal. This alters the distribution of the protein by preventing translocation into the nucleus, so the pathogenic variant accumulates in the cytosol [303]. Additionally, nonsense-mediated decay is impaired, therefore cytosolic accumulation of protein promotes the formation of cytoplasmic inclusions via an alternate mechanism of aggregation [303]. This could suggest that failed activity in the nucleus might play a greater role in phenotype than the stress granules themselves.

### 9.4. Autophagy Dysregulation

HNRNPA2B1 has also been shown to mediate autophagy in breast and ovarian cancer [319,320]. In breast cancer, m6A activity of hnRNPA2B1 decays ATG4B mRNA, which codes for a cysteine protease that converts LC3 to LC3-I. In this way, hnRNPA2B1 is able to inhibit autophagy by preventing the development of the autophagosome [320]. Downregulation of HNRNPA2B1 activates autophagy and simultaneously reduces the proliferative capacity of breast cancer cells [320]. In ovarian cancer, hnRNPA2B1 promotes tumorigenesis by interacting with NUF2 to activate mTOR, inhibit autophagy, and promote proliferation [319]. Knocking down either NUF2 or hnRNPA2B1 inactivates mTOR, activates autophagy, suppresses proliferation, and promotes apoptosis [319]. In summary, although most research on these genes has been conducted in cancer models, this establishes the link between hnRNPA2B1/hnRNPA1, autophagy, and cellular proliferation.

### 9.5. Critical Analysis

The cytotoxic stress granule hypothesis alone is insufficient to explain the breadth of clinical phenotypes both within and across pathogenic stress granule-associated proteins. hnRNPA2B1 and hnRNPA1 expression begin to increase in differentiating and proliferating mouse muscle satellite cells, and if these proteins are sequestered in stress granules as opposed to the nucleus, they may fail to act efficiently as myogenic splicing regulators [301]. Moreover, there is some evidence to suggest the abnormal distribution and depletion of hnRNPA2B1 and hnRNPA1 in sporadic inclusion body myositis in the absence of variants that drive stress granule aggregation [321].

We can speculate that mutating these proteins could impair differentiation by modulating autophagy in myogenesis or via alternative splicing. As we have seen, when hnRNPA2B1 is downregulated, myogenesis is impaired. Thus, a pertinent question here is as follows: if pathogenic hnRNPA2B1 cannot localize to the nucleus and transcriptional regulation is reduced, does this mimic downregulation of the protein? Further investigation could explore these pathways in relation to myogenesis.

## 10. Future Work and Implications for Myopathies

In this review, we highlight recent advances in the understanding of how myogenic differentiation is impacted in adult-onset genetic myopathy. Overall, we identify commonalities across genetically distinct diseases, suggesting that differentiation defects may be a shared feature of a broader range of myopathies. An important avenue for future investigation would be determining the role of sex in myopathy and differentiation. This is relevant due to observed clinical differences in disease severity, onset, and the inheritance of myopathies between males and females. Determining if sex influences satellite cells and differentiation could be essential for developing therapies that are effective for everyone.

Common themes that arose across genes were dysregulated autophagy/mitophagy and impaired mitochondrial function (Table 1, Figure 2). Dysregulated autophagy could either be a consequence of aberrant differentiation signaling, or it could contribute to this phenotype. Notably, the reactivation of mitophagy and autophagy ameliorates pathogenicity in *COL6A1* null mouse muscle [322,323]. Additionally, in young males with DMD, we observe reduced autophagy over time that correlates with a decrease in muscle regeneration [324]. We noted disrupted autophagic/mitophagic activity in FHL1, OPMD, DM1, GNE, DES, CAPN3, and HNRNP-related disease (Figure 2). As discussed, the crucial link between autophagy, mitophagy, and proper satellite cell activation and differentiation implies that satellite cells with defects in this area might also be impaired in their ability to functionally regenerate damaged muscle. Future research could focus on modulating autophagy using activators such as spermidine or rapamycin in these conditions to see if this rescues differentiation capacity.

### 10.1. Contribution of Myopathic Variants to Disease Phenotype

For each myopathy, it is crucial to determine whether variants exert a direct negative impact on satellite cells, or whether the impaired regenerative response is manifested by a changed microenvironment due to factors such as elevated inflammation, fatty and fibrotic infiltration, or an altered secretory profile from myofibers. While a controlled inflammatory response is critical for muscle regeneration [325], chronic inflammation, as observed in some dystrophies and myopathies, is associated with the impaired activation of satellite cells and inhibited differentiation and regeneration [326,327,328]. In FSHD, these links are established, but it is reasonable to suppose that this could be a contributing factor in other diseases. Given that definitive evidence of the mechanisms of differentiation impairment in myopathy is still lacking, this review discusses the strength of associations between differentiation and myopathic variants. Briefly, there are convincing connections to differentiation with variants in *FHL1*, *CAPN3*, *DUX4*, and *DES*. There are suggested links with variants in *DMPK*, *GNE*, *HNRNPA2B1*, and *HNRNPA1*. Again, all of these connections require further investigation to determine whether there is a direct or indirect impact on satellite cell function.

### 10.2. Expanding the List of Satellite Cell-Opathies

Aspects of impaired differentiation have been reported in relation to Dystrophin in DMD [329], and Emerin, Lamin A/C, Nesprin-1/2, FHL1, and SUN1/SUN2 in EDMD [49]. This review expands that list, highlighting FHL1 outside of EDMD, and adds *FHL1*, *PABPN1*, *DMPK*, *DUX4*, *DES*, *GNE*, *HNRNPA2/B1* and *HNRNPA1* to the list of genes that may impact muscle cell differentiation when mutated (Table 1). Interestingly, several of these are differentially regulated upon satellite cell activation, with some being directly regulated by Pax7 [45] (Table 2). This affirms their ties to myogenesis. Mechanisms responsible for impaired differentiation have been proposed in some diseases, but in others, there is currently only a suggested link. Efforts to further unravel specific defects in differentiation could provide therapeutic targets and deepen our pathological understanding of these devastating diseases.

Given the extensive list of genes above, it is plausible that with the study of other genetic myopathies, similar differentiation deficits can be identified as a common feature of the pathogenesis. One interesting candidate for future study is *MATR3*. Pathogenic variants in *MATR3* can result in myopathy and ALS [330]. Interestingly, it represses *DUX4* [158], coimmunoprecipitates with hnRNPA1 and hnRNPA2B1 [331], as well as PABPN1 [332]. MATR3 affects the differentiation of iPSCs and neurons [333]. Another potential gene of interest is *TIA1*, a splicing regulator which is associated with Welander Distal Myopathy (WDM) [334,335]. *TIA1* knockout in HeLa cells and mouse embryonic fibroblasts revealed its role in regulating differentiation and promoting proliferation [336,337,338]. TIA1 is differentially regulated during satellite cell activation [45] and its role in stress granule formation has been linked to autophagy [339]. Supporting this, expression of the WDM variant in HEK293FT cells altered stress granule dynamics, increased autophagic activity, and apoptosis [340], whereas knockdown altered autophagosome formation and led to apoptosis [341]. These studies hint at MATR3 and TIA1’s potential role in regulating differentiation. Their links to other myopathic proteins and autophagy tie into the overall themes of this review. Myogenic differentiation is an intricate and complex process involving many proteins, where disruption of any part could impair it and contribute to muscle pathology. Overall, there is sufficient evidence to support exploring differentiation capabilities in other genetic myopathies, broadening the scope of diseases that may fall under the category of satellite cell-opathies.

## Figures and Tables

**Table 1:** Overview of myopathies with impaired differentiation. Prominent etiological features are shared, including age of onset, mode of inheritance, and phenotypic variability. We also briefly summarize which aspect of differentiation is affected by myopathic variants, and how autophagy or mitophagy behaves differently in disease conditions.

Disease	Gene	Onset Age	Inheritance	Phenotypic Variability	Differentiation Aspect Affected	Autophagy/Mitophagy
FHL1opathy	*FHL1*	Early onset to adult	XMPMA, EDMD—X-linked recessive SPM—X-linked dominant RBM—Autosomal dominant	High—ranges from reducing body myopathy to Emery–Dreifuss phenotype	Fusion impaired by reduced expression or MRFs	Autophagosome assembly defects, possible increased mitophagy
OPMD	*PABPN1*	Adult onset	Autosomal dominant	Low. Ptosis and dysphagia with variable late limb involvement	Fusion impaired by MRF sequestration in the nucleus	Possible autophagosome assembly defects
DM1, DM2	*DMPK*, *CNBP*	Congenital to adult	Autosomal dominant	Very high—multisystem disorder with variable severity	Reduced expression of MRFs, possibly due to mRNA destabilization	Increased autophagic flux, decreased mitophagy
FSHD	*DUX4*	Adolescent to adult	Autosomal dominant	High—ranges from asymptomatic carriers to severe disease	Suppression of MRF expression, as well as broad transcriptional dysregulation	
GNEM	*GNE*	Early adulthood onset	Autosomal recessive	Moderate—distal onset and consistent sparing of the quadriceps	Reduced expression of MRFs	Reduced autophagic activity
Desminopathy	*DES*	Adult onset	Primarily autosomal dominant	High—cardiac and respiratory involvement are variable	Desmin may regulate MRFs and variants may impair satellite cell migration	
Calpainopathy	*CAPN3*	Adolescence to adult	Primarily autosomal recessive	Moderate—proximal onset with scapular winging	Impaired fusion, MRF expression, and myonuclear positioning	Impaired autophagosome clearance
IBMPFD, LGMD1E	*HNRNPA2B1*, *HNRNPA1*	Adult onset	Autosomal dominant?	High—multisystem with inclusion body myopathy, Paget’s disease, dementia	RNA processing defects	Directly regulates autophagy activation through LC3

**Table 2:** Common features of myopathic genes. Common themes of genetic myopathies considered in this paper are listed, including transcription factors whose behavior has been reportedly altered in the disease environment. Importantly, the “-” symbol denotes areas where it has not been reported how the myopathy gene and cellular function affect each other, with the “+” symbol indicating a reported relationship.

Gene	Regulated by PAX7	Dysregulated Autophagy	Differentially Expressed During Satellite Cell Activation	Myogenic Transcription Factors Affected
*FHL1*	+	+	+	MYOG, NFAT
*DUX4*	-	-	+	MYOD
*GNE*	+	+	+	PAX7, MYOD1, MYOG
*DES*	-	+	+	MEF2C, MYOD1, MYOG
*HNRNPA2/B1*	-	+	+	
*DMPK*	-	+	+	PAX7, MYOD1, MYOG
*PABPN1*	-	+	-	PAX7, MYOD1, MYOG, MYF5
*CAPN3*	-	+	-	PAX7, MYOD1, MYOG, NFAT

## Data Availability

No new data were created or analyzed in this study. Data sharing is not applicable to this article.
